# Radical rubrics: implementing the critical and creative thinking general capability through an ecological approach

**DOI:** 10.1007/s13384-022-00521-8

**Published:** 2022-04-20

**Authors:** Dan Harris, Kathryn Coleman, Peter J. Cook

**Affiliations:** University of Melbourne, Melbourne, Australia

**Keywords:** Creativity, ITE, Critical and creative thinking, Rubrics, Creative ecology, praxis

## Abstract

This article details how and why we have developed a flexible and responsive process-based rubric exemplar for teaching, learning, and assessing critical and creative thinking. We hope to contribute to global discussions of and efforts toward instrumentalising the challenge of assessing, but not standardising, creativity in compulsory education. Here, we respond to the key ideas of the four interrelated elements in the critical and creative thinking general capability in the Australian Curriculum learning continuum: inquiring; generating ideas, possibilities, actions; reflecting on thinking processes; and analysing, synthesising and evaluating reasoning and procedures. The rubrics, radical because they privilege process over outcome, have been designed to be used alongside the current NAPLAN tests in Years 5, 7 and 9 to build an Australian-based national creativity measure. We do so to argue the need for local and global measures of creativity in education as the first round of testing and results of the PISA Assessment of Creative Thinking approach and to contribute to the recognition of creative thinking (and doing) as a core twenty-first century literacy alongside literacy and numeracy.

This paper promotes the inclusion of critical and creative thinking in Initial Teacher Education (ITE)—and in turn, school-based education—through the development and use of process-based creative inquiry (PBCI) rubrics. We propose that creative inquiry rubrics are radical in their attention to teaching, learning and assessing processes over outcomes. We advocate for a process orientation as an antidote to the continuing standardisation of creativity measures, most recently seen in the incoming PISA creative thinking test (OECD, 2019). Within such a creative ecological approach (Harris, 2016a), the use of PBCI rubrics is underpinned by curriculum *as* praxis (Grundy, 1987), where the practice of becoming a teacher is intertwined with the pre-service teachers' experience of being creative and critical thinking learners. We are also drawing on Freirean praxis pedagogy beliefs (1972), where reflection and immersion in the field connect theoretical underpinnings explored in initial teacher education courses with their practical implementation during professional experiences. The radical rubric approach aims to provide pre-service teachers with meaningful, authentic experiences in transforming creative and critical education so that they are equipped to design and develop meaningful, authentic critical and creative learning experiences in their future schools and classrooms. ITE programs are encouraged to integrate critical and creative inquiry activities into their cornerstone and capstone units with these radical rubrics to prepare graduates to contribute to the education sector's broader creative ecology. This approach is timely as we transition from pandemic pedagogies to endemic practices.

## Positioning critical and creative thinking in initial teacher education

Australian ITE programs are complex programs focussed on learning about teaching practices through the study of curriculum and pedagogy. These programs are developed through well-defined accredited learning designs, underpinned by curriculum, policy, educational theories and pedagogical practices in conjunction with school-based work-integrated learning (WIL) placements to prepare pre-service teachers for success. To achieve this, ITE programs are responsive to multiple reforms: professional regulatory bodies such as the Australian Institute for Teaching and School Leadership (AITSL) and the Australian Curriculum, Assessment and Reporting Authority (ACARA), including recent shifts in the wake of the COVID-19 pandemic, and compliance standards that are enforced through state and commonwealth government authorities. Higher education providers must meet standards from authorities in their home state to enable graduates to register as teachers. University courses are mapped against program standards set by AITSL to ensure that pre-service teachers are classroom ready for the challenging and diverse educational contexts they will encounter.

Reforms and systemic stresses affect early-career graduate teachers' creativity and teaching practices. They are deterred by embedded school practices rather than developing and designing cross-cutting innovative, curious and collaborative future-focussed learning and teaching. Reform pressures are increased by systemic stressors that focus on performance, high-stakes assessment, national testing results and the need to meet national and international benchmarks. Over the last few years, Australian reforms have been directed by the following vision documents: Teacher Education Ministerial Advisory Group Report *Action Now – Classroom Ready Teachers* (2014); compulsory testing for teachers entering the profession, implemented as the Literacy and Numeracy Test for Initial Teacher Education (ACER, 2016); *National Review of Teacher Registration* (AITSL, 2018); and current Parliamentary reviews such as the *Status of the Teaching Profession* (Parliament of Australia, 2019). While ITE programs vary institutionally, they are all limited by insufficient time to effectively deliver a coherent curriculum that is “taught, assessed and practiced” (AITSL, 2019) alongside strategies for integrating new workplace and socio-cultural skills like creativity. The use of more flexible and process-focussed assessment tools in ITE (provided ITE programs offer adequate learner and teacher experience integrated into their units) can positively influence school change, simultaneously promoting creative environments in classrooms and across whole schools, once ITE graduates find employment.

## Developments in the Australian context

In Australia, the impetus to foster creativity and innovation, and develop critical thinking skills and creative capacities was at the forefront of *The Melbourne Declaration on Educational Goals for Young Australians* (MCEETYA, 2008). More than a decade on, this can still be seen as a significant turning point in the national agenda toward valuing creativity in Australian education, as indicated in the *Alice Springs (Mparntwe) Education Declaration* (2019). The need for critical and creative thinking is well established by ACARA (2016) as a general capability to be integrated across the curriculum continuum. AITSL (2019) identifies that critical and creative thinking is a teaching strategy for effective teaching and learning to foster confident, creative and innovative young Australians. These shifts signal the growing complexity of teacher responsibilities for developing teaching and assessment skills in critical and creative thinking to design learning for unknown futures.

National Australian reviews of creative and cultural education, and employment strategies (Flew & Cunningham, 2010; Harris, 2014; Harris, 2016a, 2016b a&b; Harris & Ammerman, 2016) have synthesised the interrelationship between education practice and the need to develop creative dispositions such as inquisitiveness, persistence, imagination and collaboration in student learning. It has been further argued within ITE programs and professional teacher/school practices that ecological perspectives via whole-school strategies and audits improve professional teacher practice (Richardson & Mishra, 2018). The *Australian Government's Standing Committee on Employment, Education and Training's Inquiry into innovation and creativity: Workforce for the new economy* (Parliament of Australia, 2016) was created to ensure that “Australia's tertiary system—including universities and public and private providers of vocational education and training—can meet the needs of a future labour force focussed on innovation and creativity” (n.p). These developments in the Australian national context were mirrored globally (Beghetto, et al., 2014; Chiam, et al., 2014; DOET, 2014; Lassig, 2019) pre-pandemic and indicate a groundswell of attention to creativity education and work readiness that drives the need for further development in this area as we reimagine school and education for the future.

ITE programs synthesise both the Australian Curriculum and each state or territory's local curriculum adaptations (GWA, 2018; QLD Government, 2018; NESA, 2018; VCAA, 2016). Apart from providing the general blueprint, the Australian Curriculum provides seven general capabilities encompassing knowledge, skills, behaviours and dispositions. Critical and Creative Thinking is one of the capabilities through which students “learn to generate and evaluate knowledge, clarify concepts and ideas, seek possibilities, consider alternatives and solve problems” (ACARA, 2016). Schools expect graduate teachers to deliver these capabilities through an integrated curriculum and inter-, multi- and transdisciplinary approaches, which we argue should be explicitly taught and commenced in ITE programs if they are to be successful. The co-authors have significant experience in teaching disciplinarily and have integrated these practices from an interdisciplinary epistemological approach, which we offer as part of the radicalising of the curriculum.

The ITE provider's challenge is to locate appropriate space within their complex teacher education programs to include all capabilities while scaffolding ways to design integrated learning and develop inter-, multi- and transdisciplinary knowledge and skills (Moss, et al., 2019). Including the capabilities as part of professional experience units provides practical examples of how to implement the capabilities and the associated pedagogical knowledge for learners in their future classrooms. We assert that engaging deeply in the capabilities within the discipline and curriculum units as both learners and teachers enables greater interaction of those capabilities through two-way pedagogies (Learning Policy Institute & Turnaround for Children, 2021) as pre-service teachers themselves learn through creative inquiry methods “to find out what students are thinking, puzzling over, feeling, and struggling with” (Darling-Hammond, 2016, p. 86). A PBCI rubric that troubles perceived notions of creativity and presents an innovative approach to developing critical and creative thinking, appraisal and assessment qualities in ITE students would assist in achieving this transformation. All the general capabilities, including Critical and Creative Thinking, are noted on each state's syllabus websites, only some of which have been updated to align with the Australian Curriculum (all of which continue to be fluid documents). The states that have adopted these capabilities under the banner of learning *across* the curriculum, provide limited direction for inclusion outside of the content descriptions in some subjects and short elaborations on the ACARA site.

The inclusion of Critical and Creative Thinking in the Australian Curriculum has created an opportunity to further future-focussed learning that allows for transferable skills in a curriculum for both graduate teachers and their students. “In the Australian Curriculum, general capabilities are addressed through the learning areas and are identified where they offer opportunities to add depth and richness to student learning” (ACARA, 2016). Teaching a curriculum of the future requires skills and capabilities (Reeves, 2021), necessary in the “fourth industrial revolution” (Farrell & Corbel, 2017) and within post-pandemic pedagogies (McCarty, 2020) such as play, problem-solving, creative thinking, collaboration and digital skills. These transferable cross-cutting skills include a range of multimodal literacies (Walsh, 2010) and capabilities often referred to as “soft skills” (Lucas et al., 2013). But how do early-career teachers develop and maintain the ability to design disciplinary future-focussed creative learning and teaching? Where do early-career teachers develop their curriculum integration skills and capacities as practitioners? Arguably, these are acquired through practising over time and found in integrative disciplinary knowledge domains to support graduate teachers as learners as they traverse disciplinary and inter-, multi- and transdisciplinary skills and knowledge. The authors believe that this must begin in their initial teacher education.

## Initial teacher education (ITE) and creative ecologies

A creative ecology approach (Harris, 2016a) in ITE provides a space for learning about 'curriculum as praxis' (Grundy, 1987) and for Critical and Creative Thinking to be nurtured collectively and collaboratively rather than individually through a praxis pedagogy (Arnold & Mundy, 2020). Transferable, integrated and inter-, multi- and transdisciplinary skills are developed through inquiry-based learning (Magnussen et al., 2000) that allow for communication, creativity, problem solving, negotiation, teamwork, reflection, empathy and knowledge that cuts across disciplinary silos (Barnes & Shirley, 2007).

As a team of creative educators, we have worked with Harris' creative ecology (2016b) to develop creative inquiry-based learning in a similar holistic, collaborative and creative methodology, focussed on building creative skills across educational sites and communities. This practice-related research is underpinned by Harris' body of work (for example, Harris, 2016b) in fostering creativity in schools and communities. As such, we are led by a belief that pre-service and early-career teachers are central to generating and opening opportunities for creative ecologies within the teaching profession as they negotiate new epistemic cultures (Knorr Cetina, 2007). Our research is driven by a desire to create radical changes in education through a curriculum *as* praxis, starting within a critical praxis inquiry model of learning in ITE (Arnold, et al., 2012). As Grundy (1987) asserts, “the curriculum is not simply a set of plans to be implemented, but rather is constituted through an active process in which planning, acting and evaluating are all reciprocally related and integrated into the process” (p. 115). Pre-service teachers' ways of knowing about critical and creative thinking are bound by their experiences and skills in instructional strategies and assessment design within these disciplinary knowledge spaces rather than through practice as learners.

**Fig. 1:**
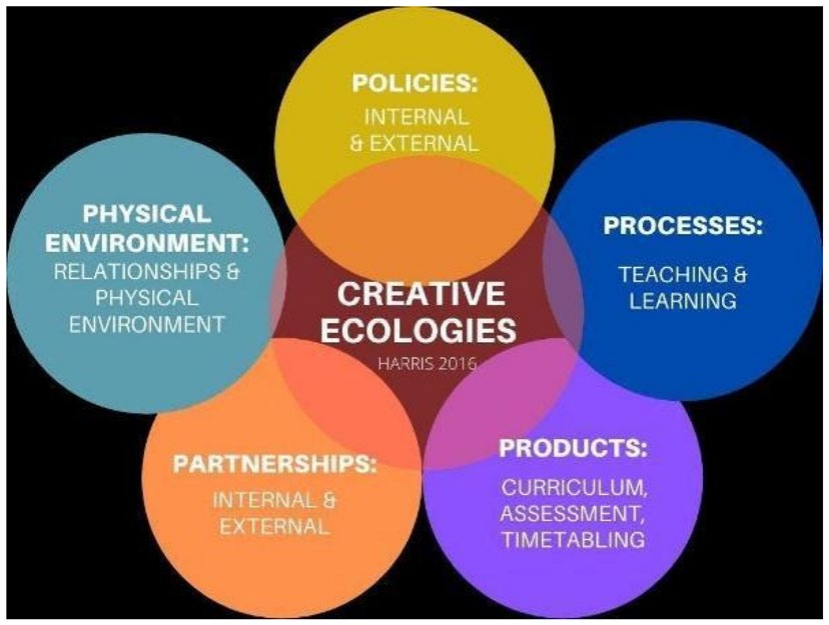
Creative ecology model (Harris, 2016b)

To intervene, we propose that Critical and Creative Thinking as a general capability is explicitly implemented within ITE programs through the praxis inquiry model of learning that enables pre-service teachers to make explicit links between practice and theory as both learners and teachers. We know it can be challenging for schools and teachers to implement this general capability, as critical thinking continues to predominate over creative thinking, often because of preconceived disciplinary differences. The essence of creative thinking is considered foundationally, often becoming an afterthought. Similarly, in ITE programs, rather than focussing on the design of the teacher education program and curriculum planning for explicit creative thinking possibilities, creativity and its possibilities remain dependent upon individual teacher educators' comfort or ability levels. Through praxis inquiry-based learning, we propose that our collaboratively developed PBCI rubric exemplar can serve as an agentic 'two-way' pedagogical tool for pre-service teachers as learners, and in-service teachers and students in schools to construct and organise knowledge about Critical and Creative Thinking. The PBCI rubrics become essential parts of the Creative Education Toolkit that connects to Harris' Creative Ecology model (Harris, 2016b), including the Creativity Index, Whole-School Creativity Audit, Top 10 Creative skills and capacities and the Creative Ecology model (Fig. 1).

The Mitchell Institute's (2016) position paper on teacher education reform mentions creativity once and offers no practical way forward, either in schools or in university ITE programs:

Teachers...are integral to developing the capabilities of young people. Not only do teachers need to be able to develop students to have inquiring minds that can think critically and creatively, but these learning dispositions are critical for teachers to possess. (p. 3)

The Practice Principles for Excellence in Teaching and Learning (2017) in Victorian schools posits that “Teachers design learning programs to explicitly build deep levels of thinking and application. This is evident when the teacher: models and develops students' critical, creative and higher order thinking skills” (p. 22). Our collaborative work is based on this premise.

It began pre-pandemic with designing and developing a PBCI rubric to support and enable ITE students to contribute positively to the creative ecologies in their creative educational ecosystems, including placement schools and future employment sites. By focussing on the inter-relationships between teaching, learning, practices and assessing for Critical and Creative Thinking, we can avoid definitional skirmishes that frequently occur in disciplinary debates and highlight creative thinking skills. Therefore, we make it clear that ITE programs need to demonstrate how they teach, practice and assess Critical and Creative Thinking through an inquiry-based learning model that can be integrated into and across all disciplinary cultures and practices in education (including in the goal of transdisciplinary work). Australian ITE programs should better reflect the changing global educational landscapes that recognise critical and creative thinking as central to learning, teaching and assessment to ensure success-ready graduates in the pandemic and endemic.

Our collaborative ecological approach offers ITE pre-service teachers experience in considering both the theoretical underpinnings of the Critical and Creative Thinking general capability and practical, implementable strategies for approaching teaching, learning and assessment on their professional experience placements to combat the conflation of *critical* with *creative* as problematic. Central to the Creative Education Toolkit are the radical rubrics, designed to align with the NAPLAN testing years, scaffolding the skills to participate in and contribute to, developing a robust creative ecology within their future schools.

## Harris' creative ecologies

Harris' formulation of a creative ecology model includes five domains that address elements in all areas of learning communities. Following Amabile and et al.'s (1996) development of valid ecological measures of creativity in workplace contexts, the Harris creative ecology heuristic follows a desire for “assessment of this complex interaction between a person's creativity and the environment” (Harris, 2016b, p. 85), in contrast to traditional approaches to fostering creativity which remains fixed solely on the individual. By drawing on Amabile's Work Environment Inventory, which assesses workplace environmental factors that are most likely to influence the expression and development of creative ideas, the Harris creative ecology model lends itself to a more environmental, collective approach to fostering creativity within the school (or any) community. This includes students, teachers, school leaders, administrators, practices, built and natural environments in and beyond the classroom, and appears in social, cultural, material and virtual spaces where teachers and students interact for the purposes of learning.

Approaching creativity in education as an ecology (de Bruin & Harris, 2017; Harris, 2018) engages learners and teachers in practices stimulated by relationships and interactions within their micro, macro and meta-worlds (see Fig. 1). Creative thought results from the cognitive, physical, emotional and virtual interaction between people, problems, situations and experiences triggered through affordances that allow such connectivity (McWilliam, 2010). A creative ecology demands a systems approach in which all elements of the ecology work in relation to one another, none in isolation. Harris' creative ecologies and the associated literature offer a beneficial framework for designing adaptable Creative Educational Toolkits.

Traditional assessments of creativity in education were primarily rooted in individual tasks of giftedness, talent and psychometric measures (Eysenck, 1996; Mayer, 1999; Runco & Mraz, 1992; Torrance, 1974). However, the creative ecologies approach recognises how an education site's people, practices and places are intertwined and connected, working in, out of and through each other—creating the *conditions* for creativity to thrive, rather than focussing on individual attributes. These ecological connections and conditions enable and allow each entity within the ecosystem to develop through interactions and flows, permeating barriers and discarding false binaries of 'inside' and 'outside', 'individual' and 'collective' activities. One benefit of approaching thinking ecologically is that it provides a framework to support learners and learning, alongside beginning teachers, through attention to the whole-school site, system and community.

A creative ecology model in ITE prepares teachers as future ready by learning about creative practice through practice (Darling-Hammond, et al., 2005). Underpinned by Harris' (2016b) *Creativity and Education*, the creative ecologies approach fosters creativity through an interconnected, iterative approach across professional and disciplinary communities within the school and throughout the sector. Implementing this approach at the beginning of ITE programs, where creative and critical teaching and learning becomes a component of pre-service teachers' core work, centres creativity training regardless of subject or developmental stage (early childhood, primary or secondary). This cyclic program design creates an evaluative feedback loop where pre-service teachers move into the schooling sector with evidence of teaching, practising and assessing the Critical and Creative Thinking general capability as learners themselves.

Because the ecology model requires collaboration to provide the right conditions for integrated creative change, authentic inquiry-based learning designs could be implemented during placements, with mentoring from experienced teachers and university lecturers. The “creative ecological approach to whole-school change” (Harris, 2016b, p. 8) models the ACARA speculative and integrative Critical and Creative Thinking learning continuum that begins with imagination and wonderment. The capacity to learn, create and innovate combined with the capacity to initiate and sustain change are attributes that transfer across contexts. By creating the conditions for teachers to continue to develop critical and creative thinking skills as learners through practice, they adapt to a continually changing and dynamic profession. We believe that developing pre-service teachers' creative and critical thinking skills and capacities through an ecological approach demands effective collaboration, enhancing the school community's unity and providing peer-sustained embedded professional development as part of everyday practice.

## Radical Rubrics as important components of a diversified toolkit

We commenced this project as a group of practitioners and researchers: educators experienced in the field of Initial Teacher Education. In forging this collaborative laboratory ('collaboratory') for addressing creative assessment, it was necessary—as a starting point—that we held similar beliefs about the influence of ITE and shared values about the transformative power of creativity. A deep understanding of creativity in education was also common amongst the co-authors, all having employed creative approaches in education at various levels and across multiple learning areas. Approaching this work as both artists and educators was integral to understanding criticality and creativity, inter-, multi- and transdisciplinary and diverse approaches to creativity within the curriculum and beyond.

The remainder of this article explains how we as a collaboratory developed these radical rubrics against the Australian Curriculum General Capability against the USA Common Core Standards (CCSS) and the OECD Learning Framework 2030 (OECD, 2018), which share considerable overlap in identifying a need for fostering creative capacities.

While the Australian standards set grade-specific goals, they do not define how the standards should be taught or which materials should be used to support students, and the supports that effectively enhance creative and critical thinking through CCSS aligned Creativity & Innovation Rubrics (Kingston, 2018) have also been considered. The OECD Learning Framework 2030 (OECD, 2018) articulates learner qualities beyond epistemic and procedural knowledge, and cognitive and social skills. That schema reinforces the need to develop attitudes and values that (in preparing for 2030 and beyond) should enable learners to:

…think creatively, develop new products and services, new jobs, new processes and methods, new ways of thinking and living, new enterprises, new sectors, new business models and new social models. Increasingly, innovation springs not from individuals thinking and working alone, but through cooperation and collaboration with others to draw on existing knowledge to create new knowledge. The constructs that underpin the competency include adaptability, creativity, curiosity and open-mindedness. (OECD, 2018, p. 5)

Our inquiry-based learning model integrates elements of the CSSS, the OECD Future of Education and Skills 2030 (2018) and the Australian Curriculum. We have developed and designed radical rubrics for teaching, practising and assessing processes, and to instrumentalise the key ideas of the four interrelated elements in the Critical and Creative Thinking learning continuum:

- Inquiring
- Generating
- Reflecting
- Analysing

The radical rubric design was developed to be used in alternate years from the current NAPLAN tests in Years 5, 7 and 9. We link this system of creativity measurement to Australian NAPLAN tests to build an Australian national creativity measure alongside the literacy and numeracy measures in the current NAPLAN testing regime. In December 2022, the PISA 2021 Assessment of Creative Thinking results will be published. They will elevate the recognition of creative thinking (and doing) as a core literacy alongside literacy and numeracy, underlining further focus on creativity assessment at a global scale (Bouchie, 2019).

**Fig. 2:**
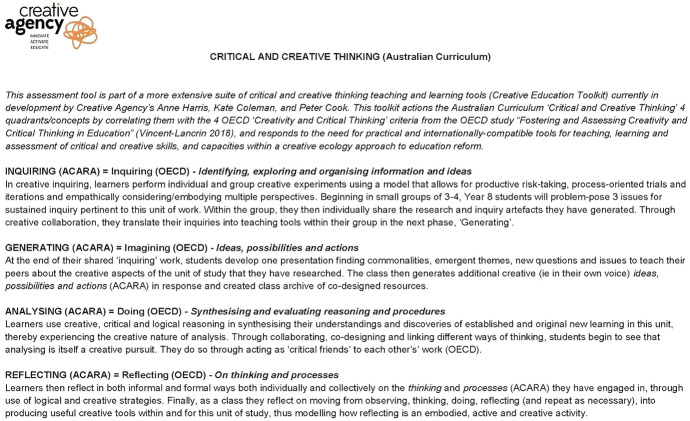
Description of assessment rubric quadrants 10.26188/14736660

While we recognise that tensions exist across Australian states between implementing a national curricular capability into localised state agendas responsible for implementation, we have designed this overarching assessment strategy through PBCI rubrics (Fig. 2) useful for schools across the nation. The radical rubric and Creative Education Toolkit approach reflects our belief that the most effective way to design and develop Critical and Creative Thinking as an essential component of all learning in Australia is by aligning with yearly national assessment years via NAPLAN, and within ITE programs, where pre-service teachers develop knowledge and experiences of curriculum, pedagogy and policy—in addition to the PISA tests, which only occur every three years for member nations.

This radical rubric may be used as a capstone or foundational tool in ITE programs to foster assessment of creativity through practice; however, it can also be used individually or collectively within a school- or university-based ecological model. As Moss, et al. (2019) agree, the “general capabilities need to be targeted explicitly within the assessment criteria or learning goals when integrated approaches are used” (p. 35). By offering this more flexible approach to creativity assessment, this rubric allows students, pre-service teachers and early-career teachers to engage in meaningful creative inquiry-based learning that has both individual and collaborative benefits for the whole-school creative ecology.

The radical rubrics within the Creative Education Toolkit act as an iterative tool through which learners design, develop and review their inquiry. The rubric as an agentic tool allows the merging of learning experiences with ongoing engagement and collaboration. It offers learners (teacher educators and pre-service teachers and in-service teachers with students) to construct and organise knowledge themselves, engage in detailed research, inquiry, writing and analysis, and communicate effectively to audiences. Leadbeater (2008) argues that the successful reinvention of educational systems worldwide depends on transforming pedagogy and redesigning learning tasks. Promoting learner autonomy and creativity through inquiry learning within ITE programs is part of the solution. The Mitchell Institute (2016) note this approach:

…highlights the increasing duality of the modern teacher – that of both teacher and learner. It also suggests that 21st century teachers will be unable to navigate the modern educational workplace without the skills and dispositions that enable them to focus on their own learning and improvement. (p. 3)

## Proposition: a model for teaching, practising and assessing Critical and Creative Thinking

The next section introduces a radical rubric that promotes teaching, practising and assessing creativity and critical thinking in ways that move beyond binaries such as 'standardised' and 'creative' instead of an imaginative, empathetic and inquiry-focussed interdisciplinary assessment tool. Our 'sleight-of-hand' in offering what may at first seem like a capitulation to standardised assessment is the kind of tool that can serve both or, as Maxine Greene argued, offer an imaginative approach that can work within simple standardisation “to combat standardardization” (1995, p. 380). We focus on PBCI rubrics within the Creative Education Toolkit as common ways to explore learning design for authentic inquiry-based tasks. They can be designed to create a backward mapping of the task and offer learners a way into the processes and reflective practices involved in ideation, problem posing, visioning and wondering about things rather than focussing on a preconceived product of learning or the content. They support “teachers who recognize the important role of imagination and creative play in the learning process, [and] want to include these higher-level thought processes as part of authentic assessment” (Young, 2009, p. 74). Our rubric design offers teachers new ways to reinforce creative practices and processes learned in ITE programs that can be supplemented by ongoing professional development in schools where creativity and critical thinking become observable, teachable and assessable.

Rubrics such as this exemplar can be deployed in Years 6, 8 and 10 (the interstitial years between NAPLAN testing in Years 5, 7 and 9) as part of a networked ecological approach to fostering creativity in educational settings (Harris, 2018). Using flexible and adaptable process rubrics allows teachers and learners to negotiate creative practices across various needs and sites. The ecological approach to creativity education (reflected in the radical rubric) invites teachers, students and school leaders to foster creativity in a whole environment but interconnected manner across the entire ecosystem within which learning takes place. Teachers traditionally interpret curriculum documents and apply pedagogies to facilitate learning via the transmission of knowledge and engagement in specific activities to that subject and particularly to that individual teacher. This rubric's interconnected and cross-curricular application allows teachers and students to find connectivities between and across domains and dismantle the siloed information transfer systems that occur within prevailing strict procedural frameworks of content, resources, timelines and assessment/reporting. As a learning and teaching tool, a PBCI rubric such as ours allows for the mental and psychological linking across a whole school that enables students, teachers and leaders to think and act on ideas and situations (Cowan, 2006). As an assessment tool, the radical rubric design stimulates imagination, ideation, wondering and possibility thinking, and synthesis and integrative thinking that enables all ecology members to contribute to each school's unique creative needs and resources.

The rubric is described in Fig. 2 (10.26188/14736660) and provides the framework for implementation. The rubric relies on the inclusion of four quadrants consistent with those specified in the General Capabilities in the Australian Curriculum and cross-referenced with the criteria in the OECD study, “Fostering and assessing creativity and critical thinking in education” (Vincent-Lancrin, et al., 2019). The quadrants are inquiring, generating, analysing and reflecting. The descriptors offer clarification of how the quadrant would be demonstrated in practice. The description deliberately remains free of learning area content to encourage transferability across disciplines.

**Fig. 3:**
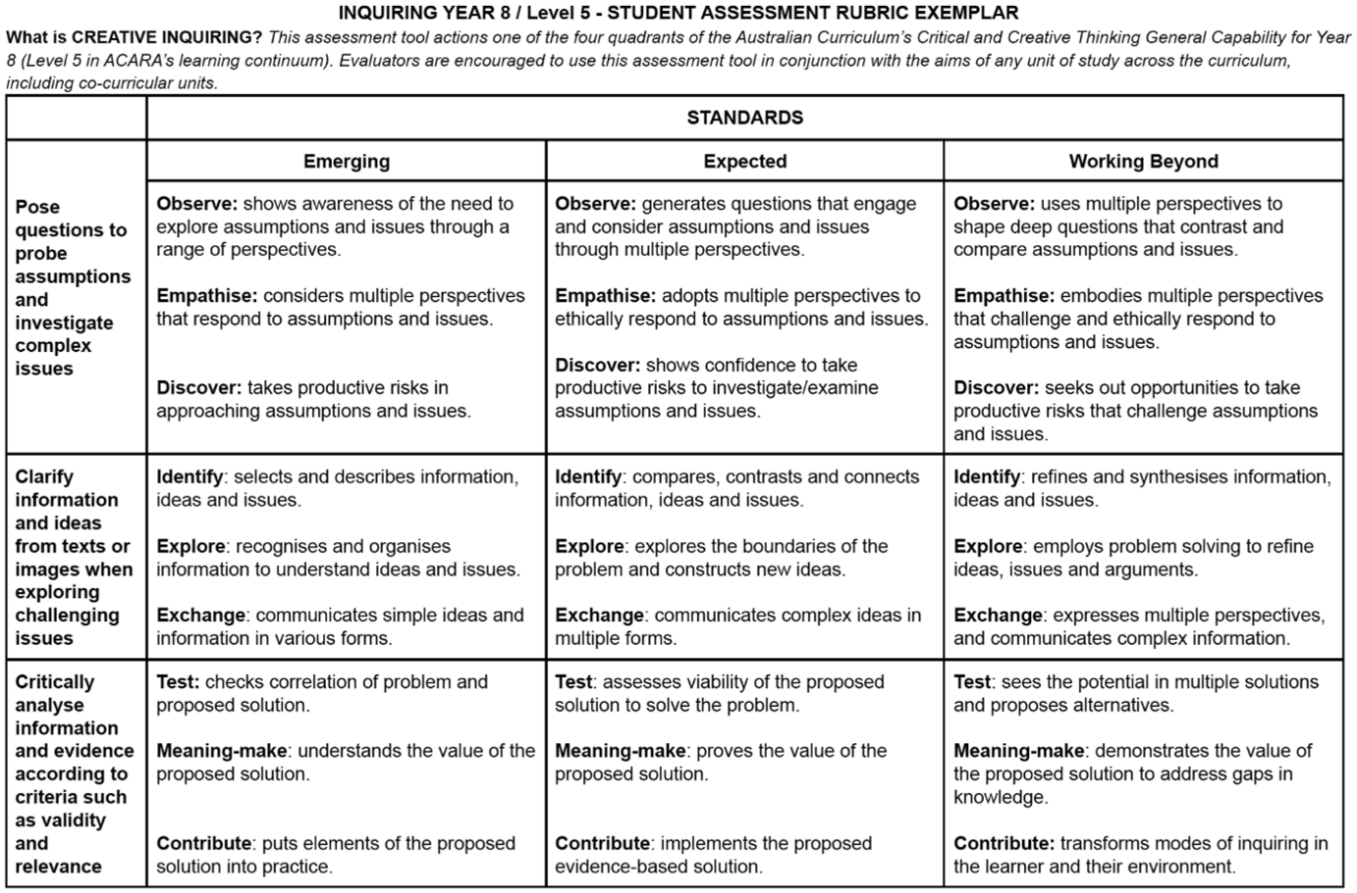
Assessment rubric standards and descriptors 10.26188/14736576

Figure 3 (10.26188/14736576) provides an example of the achievement standards for the first quadrant of inquiring. We have developed the achievement standards as suggested indicators of student levels of learning. There are three criteria presented against the standards of emerging, expected and working beyond. Each descriptor provides examples of the levels of learning achieved and outlined with an active verb to allow an evaluator to decide the level of achievement and generate appropriate feedback loops. In the context of this creative inquiry rubric, the evaluation can be conducted by a teacher or student (peer) and completed at various stages within a task.

**Fig. 4:**
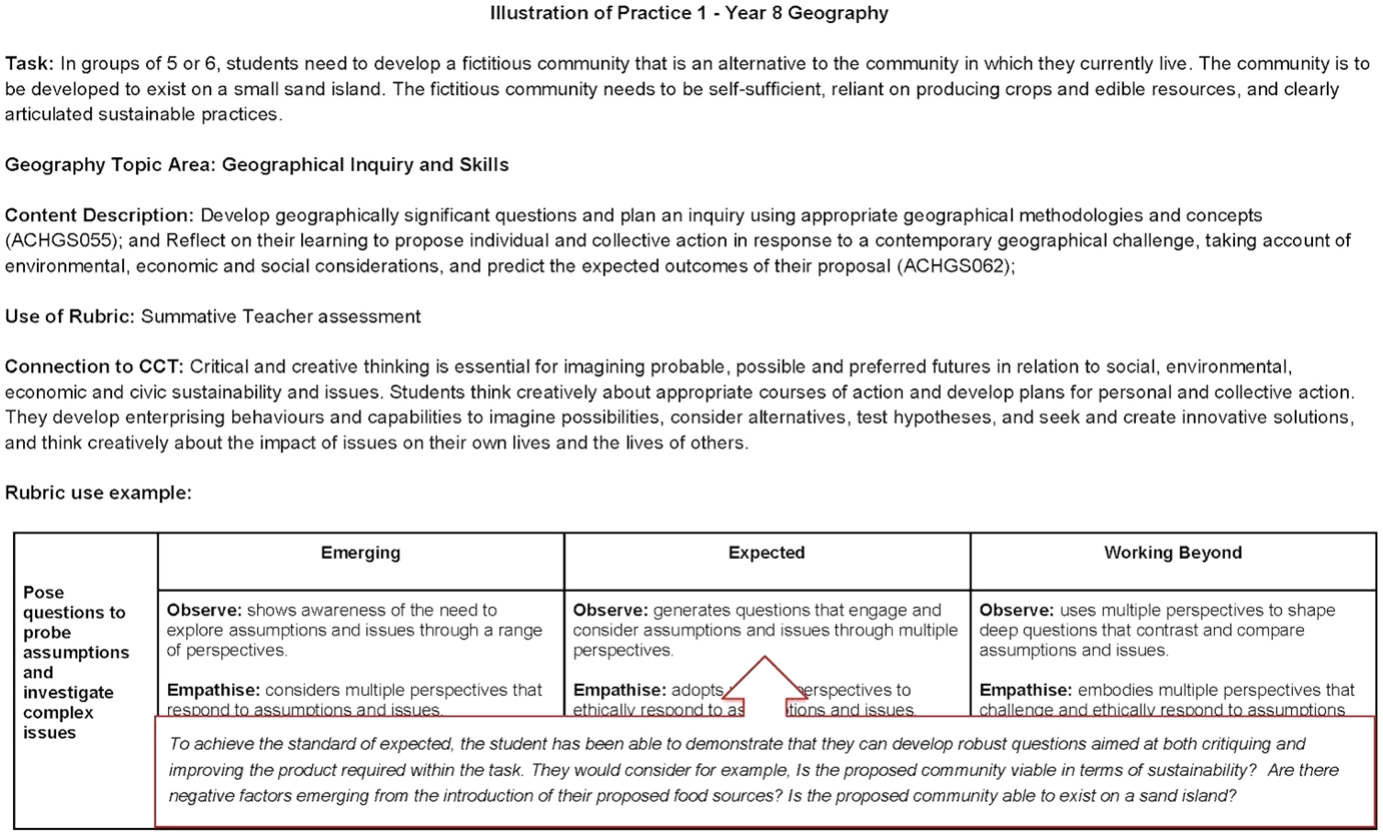
Illustration of practice 1 (year 8 geography) 10.26188/14730429

The rubric provides illustrations of practice that enable teaching staff to consider potential implementation ideas. Figure 4 (10.26188/14730429) provides an example of a Year 8 Geography task. The example provides a brief description of the assessable task and how it would align topic areas and content description. The content description in focus is derived from the Australian Curriculum. The connection to the Critical and Creative Thinking general capability is also included to highlight the existing policy documents and how these may be taught, assessed and practiced. Further description of what the achievement standard might look like is included to guide the correlation between the PBCI rubric, the task and the demonstrable critical and creative skills being assessed. Naturally, additional criteria could be incorporated based on the assessable task's localised school and class needs.

**Fig. 5:**
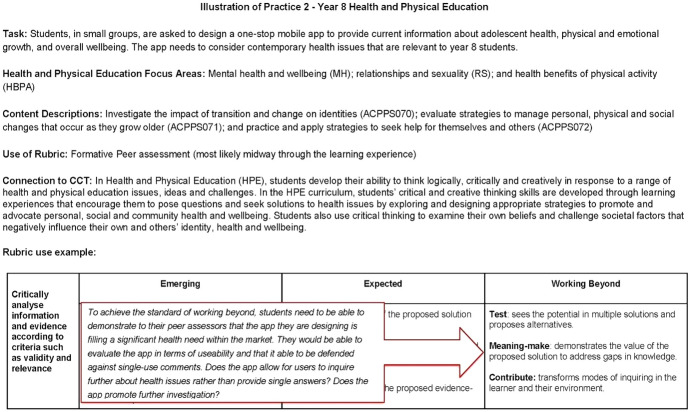
Illustration of practice 2 (year 8 health and physical education) 10.26188/14736843

The second illustration of practice is included in Fig. 5 (10.26188/14736843). It emphasises how the PBCI rubric might be used for peer learning and review. In this example, an accessible task from the learning area of Health and Physical Education is outlined within a specified focus on content found in the Australian Curriculum. Again, the relevant information about the connections to the general capability of Critical and Creative Thinking is outlined. In this example, the suggestion is that the rubric be used midway through the learning experience, with other students as the peer reviewers. The annotation on the rubric offers further exploration of what may be required to achieve a particular level of learning (in this example, 'working beyond').

## Conclusions and implications for ITE

This paper has explored how and why our collaboratory developed a flexible and responsive PBCI rubric exemplar for teaching, learning and assessing creativity to work within Harris' Creative Education Toolkit. We began this work by asking, 'how do early career teachers develop and maintain the ability to design interdisciplinary future-focussed creative learning and teaching? Where do early career teachers develop curriculum integration skills and capacities as practitioners?' This is an important time to share our praxis approach as educators worldwide face new post-pandemic challenges requiring teachers to design creative, critical, often-digital, inquiry-based learning encounters for young people. Being radical, creative and critical through a critical praxis model that challenges teaching, learning and assessment education, rather than standardising creativity in education, is needed now more than ever. Our radical rubric design provides a model for cultivating and assessing critical and creative thinking across the ecology. This kind of active feedback-feedforward loop through an inquiry model, also understood as curriculum “as praxis” (Grundy, 1987, p. 15) contributes to better practices across ITE through two-way pedagogies. Ultimately, the approach encourages ground-up creative changes in education policy.

The approach outlined in this paper suggests moving creative change in schools and ITE programs away from teacher-driven activities to co-activating problem posing as a collaborative creative practice that initiates and sustains learning through creative inquiry. The radical rubric design effectively and efficiently initiates and cultivates Critical and Creative Thinking as a general capability in ITE (and by extension into schools and classrooms). The model explored in this article is just one of the Toolkit rubrics that we propose as a set of radical interventions, which together establish more processual and accessible creative practices in learners and across whole-school creative ecologies. As such, ITE holds the potential to activate substantial and sustainable critical and creative thinking development in pre-service and early-career teachers and apply generational mindset change in all learners, by effectively developing and evolving creative communities of practice.
